# Enhanced Circularly Polarized Luminescence Activity in Chiral Platinum(II) Complexes With Bis- or Triphenylphosphine Ligands

**DOI:** 10.3389/fchem.2020.00303

**Published:** 2020-04-24

**Authors:** Qian-Ying Yang, Hua-Hong Zhang, Xue-Ling Han, Shi-Dao Weng, Yuan Chen, Jia-Li Wu, Li-Zhi Han, Xiao-Peng Zhang, Zai-Feng Shi

**Affiliations:** Key Laboratory of Water Pollution Treatment & Resource Reuse of Hainan Province, College of Chemistry and Chemical Engineering, Hainan Normal University, Haikou, China

**Keywords:** circularly polarized luminescence, platinum(II) complexes, phenylphosphine ligands, chiral enhancement, crystal structures

## Abstract

Distinct circularly polarized luminescence (CPL) activity was observed in chiral (C^∧^N^∧^N)Pt(II) [(C^∧^N^∧^N) = 4,5-pinene-6′-phenyl-2,2′-bipyridine] complexes with bis- or triphenylphosphine ligands. Compared to the pseudo-square-planar geometry of chiral (C^∧^N^∧^N)Pt(II) complexes with chloride, phenylacetylene (PPV) and 2,6-dimethylphenyl isocyanide (Dmpi) ligands, the coordination configuration around the Pt(II) nucleus of chiral (C^∧^N^∧^N)Pt(II) complexes with bulk phosphine ligands is far more distorted. The geometry is straightforwardly confirmed by X-ray crystallography. The phosphines' participation enhanced the CPL signal of Pt(II) complexes profoundly, with the dissymmetry factor (*g*_lum_) up to 10^−3^. The distorted structures and enhanced chiroptical signals were further confirmed by time-dependent density functional theory (TD-DFT) calculations.

## Introduction

Circularly polarized luminescence (CPL) materials have attracted considerable attention because of their enormous potential in 3D displays (Zinna et al., 2015; Song F. et al., 2018; Zhang et al., 2020), quantum information (Wagenknecht et al., 2010), chiroptical sensors (Carr et al., 2012; Guo et al., 2019; Wu et al., 2019), photodetectors (Yang et al., 2013; Chen C. et al., 2019) and anti-counterfeiting security (Yang et al., 2020; Yu et al., 2020). Especially these phosphorescent transition metal complexes, which have remarkable metal-center chirality, tunable emission properties, and unusually high phosphorescence efficiency, are receiving increasing interests in recent years (Han et al., 2018). Such CPL-active materials as Pt (Shen et al., 2014), Ir (Han et al., 2017; Hellou et al., 2017; Yan et al., 2019a), Au (Yang et al., 2020; Zhu et al., 2020), Cu (Jin et al., 2019; Deng et al., 2020; Yao et al., 2020), Zn (Aoki et al., 2017; Chen Y. et al., 2019) Cd (Deng et al., 2019), and Cr (Jiménez et al., 2019) complexes can exhibit various emission colors from blue to red. The dissymmetry factor *g*_lum_ (*g*_lum_ = 2Δ*I*/*I* = 2(*I*_L_ – *I*_R_)/(*I*_L_ + *I*_R_), where *I*_L_ and *I*_R_ indicate, respectively, the intensity of the left and right circularly polarized light), can reach up to 10^−2^ order. Furthermore, the rotatory strength of the transition probably leading to CPL activity can be distinctly enhanced by the spin–orbit coupling (SOC) effect of transition metals (Gendron et al., 2019). Therefore, those chiral luminescent complexes containing heavy metal atoms are likely to show a polarized emission.

Phosphorescent CPL-active Pt(II) complexes are known for their high emission quantum yield and large *g*_lum_ values, and they hold promise for use in novel optoelectronic devices. To obtain more efficient CPL materials, helicene skeleton (Shen et al., 2014; Biet et al., 2017), 1,1′-binaphthyls (Song J. et al., 2018; Song et al., 2019; Jiang et al., 2020), and other moieties were incorporated into phosphorescent Pt(II) systems, and a distinct enhancement in the phosphorescence and *g*_lum_ value can be reached. Chiral-at-metal phosphorescent Pt(II) complexes have been prepared by utilizing *trans*-spanning bipyridyl (Schulte et al., 2017) and substituted (2-thienyl)pyridine ligands (Usuki et al., 2019), displaying strong spectral responses both in circular dichroism (CD) and CPL. In addition, due to their square-planar geometry, molecules of Pt(II) complexes can form helical assemblies *via* Pt···Pt, π–π stacking, and hydrophobic–hydrophobic interactions, with a high dissymmetry factor (*g*_lum_) of 10^−2^ order (Ikeda et al., 2015, 2018; Zhang et al., 2015a; Tanaka et al., 2016; Park et al., 2019). More intriguingly, circularly polarized organic light-emitting phosphorescent diodes (CP-PHOLEDs) have been fabricated by using chiral Pt(II) complexes as the emitting layer, showing a display level brightness and a high *g*_lum_ factor (Brandt et al., 2016; Fu et al., 2019; Yan et al., 2019b).

**Scheme 1:**
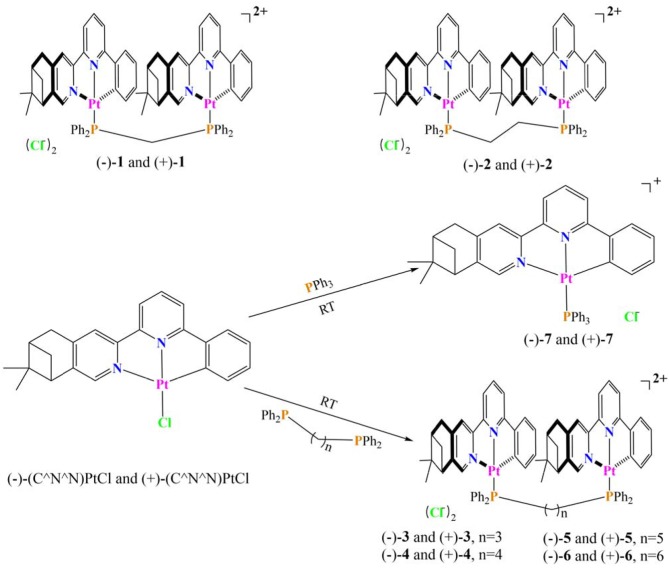
Synthesis of dinuclear and mononuclear Pt(II) complexes.

In a previous work, two couples of chiral dinuclear Pt(II) complexes, [(–)-(C^∧^N^∧^N)Pt]_2_dppmCl_2_ (–)-**1** and [(+)-(C^∧^N^∧^N)Pt]_2_dppmCl_2_ (+)-**1**, (–)-(C^∧^N^∧^N)Pt]_2_dppeCl_2_ (–)-**2**, and [(+)-(C^∧^N^∧^N)Pt]_2_dppeCl_2_ (+)-**2**, linked by bis(diphenylphosphino)methane (dppm) and bis(diphenylphosphino)ethane (dppe), were prepared (Scheme 1). Distinct CPL signals triggered by an intramolecular Pt···Pt interaction (Zhang et al., 2018) were observed. In this work, new chiral Pt(II) complexes with longer bridging ligands, [bis(diphenylphosphino)propane (dppp), bis(diphenylphosphino)butane (dppb), bis(diphenylphosphino)pentane (dpppe), and bis(diphenylphosphino)hexane (dpph)], were prepared and characterized by single-crystal X-ray crystallography. Comparisons of the chiroptical spectra to the precursors and impactors on CPL enhancement were presented.

## Results and Discussion

### Synthesis

The precursors mononuclear complexes (–)-(C^∧^N^∧^N)PtCl and (+)-(C^∧^N^∧^N)PtCl were prepared according to previous procedures (Zhang et al., 2014, 2015b). The target dinuclear and mononuclear Pt(II) complexes have been facilely synthesized though the coordination reaction between different phosphine ligands and precursors in the proper proportion at room temperature (Scheme 1). The new obtained complexes were fully characterized by NMR and MS spectra. The preparation of their enantiomers was done using the same procedure.

#### Synthesis of [(–)-(C^∧^N^∧^N)Pt]_2_dpppCl_2_, (– p3

Under an argon atmosphere, a solution (30/10 ml CH_2_Cl_2_/CH_3_OH) of (–)-(C^∧^N^∧^N)PtCl (278 mg, 0.50 mmol) and dppp (103 mg, 0.25 mmol) was stirred at room temperature for 12 h. Then, the solvent was removed under reduced pressure. The residue was washed with *n*-hexane and was further purified by recrystallization in chloroform. Lastly, green-yellow powders were obtained (65%). MS (electrospray ionization, ESI) (*m*/*z*): [M]^2+^ Calcd. for C_73_H_68_N_4_P_2_Pt_2_, 725.7; found, 726.4. Anal. Calcd. for C_73_H_68_N_4_P_2_Pt_2_Cl_2_ [**(–)-3**]: C, 57.52; H, 4.50; N, 3.68%. Found: C, 57.54; H, 4.48; N, 3.67%. ^1^H NMR (400 MHz, CDCl_3_, 298 K): δ 8.48 (s, 4H), 8.38 (s, 2H), 7.66–7.80 (m, 10H), 7.35 (m, 10H), 7.28 (t, *J* = 7.2 Hz, 2H), 7.11 (d, *J* = 7.2 Hz, 2H), 6.57 (t, *J* = 7.2 Hz, 2H), 6.38 (d, *J* = 7.2 Hz, 2H), 6.30 (t, *J* = 7.2 Hz, 2H), 6.04 (s, 2H), 3.13 (d, *J* = 14.0 Hz, 4H), 2.87 (m, 6H), 2.57 (m, 2H), 2.27 (m, 2H), 1.89 (m, 2H), 1.30 (s, 6H), 0.95 (d, *J* = 10.0 Hz, 2H), 0.43 (s, 6H). ^13^C NMR (100 MHz, CDCl_3_, 298 K): δ 162.8, 156.5, 153.8, 150.6, 147.7, 147.1, 146.6, 143.9, 137.5, 135.4, 133.5, 133.4, 133.3, 131.5, 131.0, 130.7, 130.4, 130.1, 129.5, 129.4, 129.2, 129.1, 125.4, 124.5, 120.8, 119.8, 45.3, 39.4, 39.0, 33.5, 31.2, 29.8, 27.5, 25.8, 21.4.

#### Synthesis of [(–)-(C^∧^N^∧^N)Pt]_2_dppbCl_2_, (– b4

The synthesis method was the same as the one used in preparing **(–)-3**, but replacing dppp with dppb. The product yield was 65%. MS (ESI) (*m*/*z*): [M]^2+^ Calcd. for C_74_H_70_N_4_P_2_Pt_2_, 732.7; found, 733.4. Anal. Calcd. for C_74_H_70_N_4_P_2_Pt_2_Cl_2_ [**(–)-4**]: C, 57.77; H, 4.59; N, 3.64%. Found: C, 57.76; H, 4.59; N, 3.62%. ^1^H NMR (400 MHz, CDCl_3_, 298 K): δ 8.41 (d, 2H, *J* = 8.0 Hz, 2H), 8.32 (s, 2H), 8.29 (t, *J* = 8.0 Hz, 2H), 7.65–7.75 (m, 10H), 7.32–7.41 (m, 10H), 7.28–7.31 (m, 4H), 6.75 (t, *J* = 7.2 Hz, 2H), 6.41 (t, *J* = 8.0 Hz, 2H), 6.38 (t, *J* = 8.0 Hz, 2H), 6.10 (s, 2H), 3.11 (d, *J* = 16.0 Hz, 4H), 2.82 (m, 2H), 2.59 (m, 6H), 2.29 (m, 2H), 1.92 (t, *J* = 5.2 Hz, 4H), 1.31 (s, 6H), 1.03 (d, *J* = 9.6 Hz, 2H), 0.44 (s, 6H). ^13^C NMR (100 MHz, CDCl_3_, 298 K): δ 163.2, 156.5, 154.2, 150.7, 147.3, 146.7, 146.5, 143.5, 137.4, 135.7, 133.7, 133.6, 133.1, 133.0, 131.6, 131.4, 130.9, 130.3, 129.6, 129.5, 129.2, 129.1, 125.6, 125.5, 124.6, 120.7, 119.9, 45.4, 39.5, 39.1, 33.5, 31.2, 29.9, 27.9, 25.9, 24.6, 21.5.

#### Synthesis of [(–)-(C^∧^N^∧^N)Pt]_2_dpppeCl_2_, (– p5

The synthesis method was the same as the one used in preparing **(–)-3**, but replacing dppp with dpppe. The product yield was 60%. MS (ESI) (*m*/*z*): [M]^2+^ Calcd. for C_75_H_72_N_4_P_2_Pt_2_, 739.7; found, 740.5. Anal. Calcd. for C_75_H_72_N_4_P_2_Pt_2_Cl_2_ [**(–)-5**]: C, 58.03; H, 4.67; N, 3.61%. Found: C, 58.06; H, 4.65; N, 3.59%. ^1^H NMR (400 MHz, CDCl_3_, 298 K): δ 8.64 (s, 2H), 8.62 (d, *J* = 8.0 Hz, 2H), 8.28 (t, *J* = 8.0 Hz, 2H), 7.81 (d, *J* = 8.0 Hz, 2H), 7.62–7.73 (m, 8H), 7.54 (m, 2H), 7.40–7.49 (m, 12H), 6.98 (t, *J* = 7.2 Hz, 2H), 6.73 (t, *J* = 7.2 Hz, 2H), 6.59 (d, *J* = 7.2 Hz, 2H), 6.13 (s, 2H), 3.14 (d, *J* = 14.0 Hz, 4H), 2.67 (m, 4H), 2.53 (m, 2H), 2.25–2.30 (m, 4H), 1.95 (m, 4H), 1.88 (t, *J* = 4.4 Hz, 2H), 1.29 (s, 6H), 0.96 (d, *J* = 10.0 Hz, 2H), 0.46 (s, 6H). ^13^C NMR (100 MHz, CDCl_3_, 298 K): δ 163.3, 156.7, 154.5, 151.3, 148.3, 147.5, 146.6, 143.4, 137.1, 135.2, 133.8, 133.7, 133.6, 132.1, 131.9, 131.1, 130.4, 129.8, 129.6, 129.5, 129.4, 125.9, 125.6, 125.1, 121.1, 119.5, 45.5, 39.4, 39.1, 33.5, 31.1, 29.8, 27.2, 26.4, 25.9, 21.6.

#### Synthesis of [(–)-(C^∧^N^∧^N)Pt]_2_dpphCl_2_, (– h6

The synthesis method was the same as the one used in preparing **(–)-3**, but replacing dppp with dpph. The product yield was 60%. MS (ESI) (*m*/*z*): [M]^2+^ Calcd. for C_76_H_74_N_4_P_2_Pt_2_, 746.7; found, 747.5. Anal. Calcd. for C_76_H_74_N_4_P_2_Pt_2_Cl_2_ [**(–)-6**]: C, 58.27; H, 4.76; N, 3.58%. Found: C, 57.28; H, 4.74; N, 3.57%. ^1^H NMR (400 MHz, CDCl_3_, 298 K): δ 8.66 (s, 2H), 8.63 (d, *J* = 8.0 Hz, 2H), 8.27 (t, *J* = 8.0 Hz, 2H), 7.76 (t, *J* = 8.0 Hz, 6H), 7.69 (t, *J* = 8.0 Hz, 4H), 7.53 (m, 4H), 7.45 (m, 10H), 7.03 (t, *J* = 7.2 Hz, 2H), 6.81 (t, *J* = 7.2 Hz, 2H), 6.62 (d, *J* = 7.2 Hz, 2H), 6.19 (s, 2H), 3.16 (d, *J* = 14.0 Hz, 4H), 2.72 (m, 2H), 2.58 (m, 8H), 2.26 (m, 2H), 1.90 (t, *J* = 4.4 Hz, 6H), 1.30 (s, 6H), 0.99 (d, *J* = 9.6 Hz, 2H), 0.49 (s, 6H). ^13^C NMR (100 MHz, CDCl_3_, 298 K): δ 163.3, 156.7, 154.5, 151.3, 148.2, 147.5, 146.6, 143.3, 137.1, 135.3, 133.7, 133.6, 133.5, 132.0, 131.9, 131.2, 130.4, 129.8, 129.6, 129.5, 129.4, 129.3, 125.8, 125.5, 125.0, 121.0, 119.3, 45.4, 39.4, 39.0, 33.4, 31.0, 30.5, 27.1, 26.9, 25.9, 21.5.

#### Synthesis of (–of C^∧^N^∧^N)PtPPh_3_Cl, (– P7

The synthesis method was the same as the one used in preparing **(–)-3**, but replacing dppp with triphenylphosphine (PPh_3_), and the molar ratio of (–)-(C^∧^N^∧^N)PtCl to PPh_3_ is 1:1. The product yield was 70%. MS (ESI) (*m*/*z*): [M]^+^ Calcd. for C_41_H_36_N_2_PPt, 782.2; found, 782.9. Anal. Calcd. for C_41_H_36_N_2_PPtCl [**(–)-7**]: C, 60.18; H, 4.43; N, 3.42%. Found: C, 60.20; H, 4.41; N, 3.41%. ^1^H NMR (400 MHz, CDCl_3_, 298 K): δ 8.86 (s, 1H), 8.81 (d, *J* = 8.0 Hz, 1H), 8.32 (t, *J* = 8.0 Hz, 1H), 7.87 (dd, *J*_1_ = 12.0 Hz, *J*_2_ = 7.2 Hz, 6H), 7.78 (d, *J* = 8.0 Hz, 1H), 7.58 (td, *J*_1_ = 7.2 Hz, *J*_2_ = 2.0 Hz, 3H), 7.49 (td, *J*_1_ = 7.2 Hz, *J*_2_ = 2.0 Hz, 7H), 7.02 (t, *J* = 7.2 Hz, 1H), 6.62 (t, *J* = 7.2 Hz, 1H), 6.43 (d, *J* = 8.0 Hz, 1H), 6.06 (s, 1H), 3.20 (d, *J* = 18.8 Hz, 2H), 2.53–2.59 (m, 1H), 2.28 (m, 1H), 1.91 (t, *J* = 5.2 Hz, 1H), 1.33 (s, 3H), 1.01 (d, *J* = 9.6 Hz, 1H), 0.49 (s, 3H). ^13^C NMR (100 MHz, CDCl_3_, 298 K): δ 163.5, 156.9, 156.8, 154.6, 151.5, 148.1, 147.4, 146.0, 143.5, 138.9, 135.7, 135.6, 134.8, 134.7, 132.2, 132.1, 130.8, 129.5, 129.3, 129.2, 128.9, 128.7, 128.6, 125.8, 125.5, 125.1, 121.3, 119.2, 119.1, 45.8, 39.4, 39.1, 33.4, 31.1, 26.0, 21.6.

### Crystal Structures

**Figure 1:**
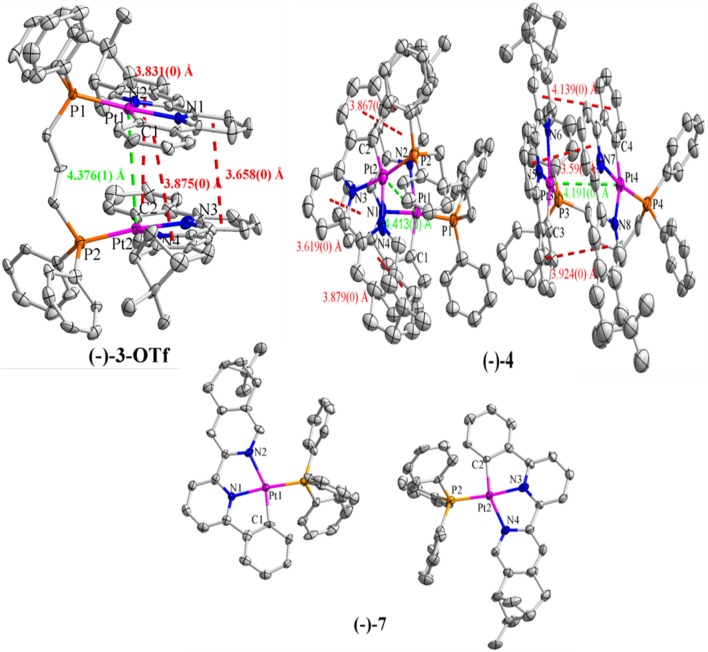
X-ray crystal structures of (–)-**3**-OTf, (–)-**4**, and (–)-**7**, with the *green dashed lines* indicating the Pt···Pt distances and the *red dashed lines* indicating the π–π distances. H atoms, solvent molecules, as well as anions are omitted for clarity.

Suitable crystals (–)-**4** and (–)-**7** for X-ray analysis were obtained by the interface diffusion of *n*-hexane into the mixed dichloromethane/acetone (*V*/*V* = 1:2) solution of respective compounds at 273 K. Although single crystals of (–)-**3** could not be obtained, green-yellow blocks of (–)-**3**-OTf were isolated *via* the same interface diffusion, where Cl^−^ was substituted by OTf^−^ through counterion metathesis. The crystal structure of (–)-**3**-OTf falls in the *P*1 space group of the triclinic system (Table 1), and only one enantiomer molecule is included in the asymmetrical unit (Figure 1). Whereas, both complexes (–)-**4** and (–)-**7** crystallize in the monoclinic space group *P*2_1_ with two separated molecules in the asymmetrical unit (Figure 1 and Table 1). The Flack values of (–)-**3**-OTf, (–)-**4**, and (–)-**7** are −0.018(8), −0.022(7), and −0.017(6), respectively, confirming the absolute configuration of the molecules.

**Table 1:** Crystallographic data of (–)-**3**-OTf, (–)-**4**, and (–)-**7**.

	**(–)-3-OTf**	**(–)-4**	**(–)-7**
Formula	C_78_H_78_F_6_N_4_O_9_P_2_Pt_2_S_2_	C_162_H_178_C_l12_N_8_O_3_P_4_Pt_4_	C_43_H_40_Cl_5_N_2_PPt
*Mr* (g mol^−1^)	1,845.68	3,614.76	988.08
Crystal system	Triclinic	Monoclinic	Monoclinic
Space group	*P*1	*P*2_1_	*P*2_1_
*a* (Å)	12.0402(10)	15.4780(4)	12.9794(3)
*b* (Å)	13.7250(11)	21.6484(4)	13.2011(4)
*c* (Å)	13.8815(13)	22.3757(4)	23.8617(6)
α (°)	68.595(8)	90.00	90.00
β (°)	64.906(9)	94.539(2)	95.244(2)
γ (°)	74.035(7)	90.00	90.00
*V* (Å^3^)	1,914.4(3)	7,474.0(3)	4,071.41(19)
*Z*	1	2	4
*T* (K)	153(2)	153(2)	153(2)
Radiation, λ (Å)	0.71073	0.71073	0.71073
*D*_calcd_ (g/cm^−3^)	1.601	1.606	1.612
μ (mm^−1^)	3.819	4.046	3.847
*F*(000)	918	3,612	1,960
Crystal size (mm^3^)	0.26 × 0.23 × 0.20	0.29 × 0.26 × 0.20	0.28 × 0.26 × 0.21
θ range (°)	2.07–26.00	2.30–29.53	2.21–27.10
Reflections measured	15,561	37,976	24,890
Unique reflections	10,849	22,792	15,192
*R*_int_	0.0404	0.0422	0.0384
Reflections with *F*^2^ > 2σ(*F*^2^)	9,558	20,357	14,217
Number of parameters	922	1,741	905
Goodness-of-fit on *F*^2^	1.044	1.042	1.067
*R*_1_ [*F*^2^ > 2σ(*F*^2^)]	0.0490	0.0581	0.0424
w*R*_2_ (all data)	0.1165	0.1543	0.1089
Δρ_max_, Δρ_min_ (e Å^−3^)	2.184, −1.230	3.493, −2.426	1.523, −1.580
Flack parameter	−0.018(8)	−0.022(7)	−0.017(6)

In the crystal structures of (–)-**3**-OTf, (–)-**4**, and (–)-**7**, Pt–N (1.98–2.17 Å), distance is slightly larger than the Pt–C (1.97–2.04 Å) bond, similar to the previously reported bond lengths of analogous Pt(II) complexes (Table S1) (Lu et al., 2002, 2004; Shao and Sun, 2007; Zhang et al., 2018). The Pt–P (2.23–2.26 Å) bond is also consistent with that in phosphino Pt(II) complexes. In the structure of complex (–)-**3**-OTf, the angles N2–Pt1–C1 (158.8(2)°) and N4–Pt2–C2 (158.7(3)°) deviate substantially from linearity due to the chelate ring strain (Table S2). Interestingly, the N1–Pt1–P1 [176.10(16)°] and N3–Pt2–P2 [173.60(15)°] angles are also slightly distorted from linearity. Even then, the angle N3–Pt2–P2 is found at 168.75(17)° in the crystal structure of (–)-**4** (Table S2), which is much smaller than the reported values in pinene-containing (C^∧^N^∧^N)PtCl, (C^∧^N^∧^N)PtPPV, and (C^∧^N^∧^N)PtDmpi complexes (Zhang et al., 2014, 2015a,b, 2016). In addition, the torsion angles between the benzene plane and lateral pyridine plane of C^∧^N^∧^N ligands have been examined, as shown in Table S3, and the angles range from 1.3 to 14.5°. It can be inferred that the Pt(II) cation situates in a more distorted square-planar coordination environment in the phosphino-coordinating system.

As shown in Figure 1, two [(–)-(C^∧^N^∧^N)Pt]^+^ segments bridged by the dppp ligand in (–)-**3**-OTf are arranged parallel to each other with a torsion angle θ (0.74°) along the Pt–Pt axis (θ defined by the angle between the Pt1–Pt2–N1 and Pt1–Pt2–N3 planes). However, [(–)-(C^∧^N^∧^N)Pt]^+^ moieties in (–)-**4** are staggered packed along the Pt–Pt axis, with θ values of 11.74° and 16.34°. Because flexibility and steric hindrance increase with the elongation of the bridging ligand, the intramolecular Pt···Pt distances observed in (–)-**3**-OTf, (–)-**4**, and (–)-**7** are outside the range (3.09–3.50 Å) predicted for an effective Pt···Pt interaction (Zhang et al., 2018). However, weak intramolecular π–π interactions (the distance between two aromatic rings: 3.5–4.1 Å) are expected in both (–)-**3**-OTf and (–)-**4** resulting from the face-to-face conformation of two [(–)-(C^∧^N^∧^N)Pt]^+^ planes. In addition, the distances for the closest intermolecular Pt···Pt contact are over 4.0 Å in (–)-**3**-OTf, (–)-**4**, and (–)-**7**; therefore, any effective intermolecular Pt···Pt interaction is absent (Figure S1).

### Absorption and Emission Properties

**Figure 2:**
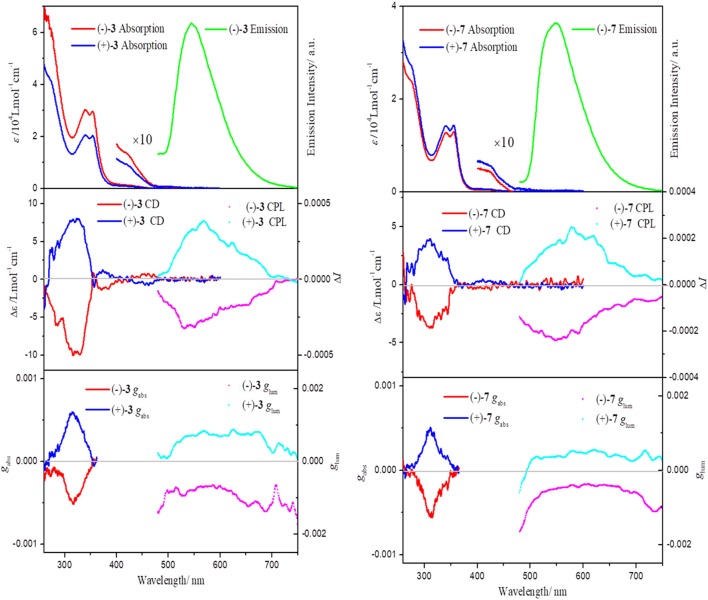
Absorption and emission spectra of (–)-**3** and (–)-**7** in CH_2_Cl_2_ (5 × 10^−5^ mol L^−1^) at *T* = 298 K, λ_ex_ = 420 nm (*top*). CD spectra in CH_2_Cl_2_ (5 × 10^−5^ mol L^−1^) at *T* = 298 K (*middle*) and CPL spectra in CH_2_Cl_2_ (10^−3^ mol L^−1^) at *T* = 298 K (*middle*). *g*_abs_ and *g*_lum_ factors are shown at the *bottom*.

As shown in Figure 2 and Figures S2–S4, all of the chiral dinuclear Pt(II) complexes show characteristic absorption bands (ε > 10^4^ L mol^−1^ cm^−1^) in the UV region similar to those of bis-(diphenylphosphino)alkane bridged dinuclear Pt(II) complexes. The mononuclear Pt(II) complex (–)-**7** also exhibits a similar intense absorption below 400 nm. According to previous studies, the intense bands (<400 nm) are attributed to intraligand π–π^*^ transitions. In addition, weak absorptions in the region of 400–450 nm are designated as a mixture of metal-to-ligand charge transfer (^1^MLCT) and ligand-to-ligand charge transfer (^1^LLCT) transitions (Lu et al., 2002, 2004; Shao and Sun, 2007; Zhang et al., 2018). From the crystal structures of (–)-**3**-OTf, (–)-**4**, and (–)-**7**, it can be found that effective intramolecular/intermolecular Pt···Pt interactions are absent and that two [(C^∧^N^∧^N)Pt]^+^ units manifest like two separated moieties (Sun et al., 2006). Correspondingly, the absorptions of all the complexes only extend to ~470 nm, which agrees well with the spectrum of (–)-**2**, demonstrating the nonexistence of metal-metal-to-ligand charge transfer transition (^1^MMLCT) (Zhang et al., 2018).

All of the chiral dinuclear and mononuclear Pt(II) complexes are highly emissive in solution. For all the dinuclear Pt(II) complexes, a broad and structureless emission band at 546 nm is seen, which resembles that of the mononuclear relative (–)-**7** (Figure S5). Similar to the absorption spectra, the emission energy of all the complexes also reflects the absence of effective intramolecular/intermolecular Pt···Pt interactions. The emission of all the complexes can be ascribed to a triplet metal-to-ligand charge transfer (^3^MLCT) excited state (Lu et al., 2002, 2004; Shao and Sun, 2007; Zhang et al., 2018). At 77 K, the emissions are significantly blue-shifted and evolve to be more structured (Figure S5), a characteristic nature for ^3^MLCT excited states. An intense emission peak and a shoulder are observed at 515 and 550 nm, respectively, and the spacing of about 1,100 cm^−1^ correlates to the characteristic skeletal stretching of the free C^∧^N^∧^N ligand.

### Chiroptical Properties

The chiroptical spectra (CD and CPL) of all the chiral Pt(II) complexes are plotted in Figure 2 (complexes **3** and **7**) and Figures S2–S4 (complexes **4**–**6**). Although the bridging ligands are different, complexes (–)-**3**, (–)-**4**, (–)-**5**, and (–)-**6** show similar CD signals with an intense negative Cotton effect at approx. 310 nm and a weak negative effect at approx. 380–400 nm in CH_2_Cl_2_ solution. Because the two [(C^∧^N^∧^N)Pt]^+^ units behave like two discrete parts, a similar CD profile is expected in the mononuclear Pt(II) complex (–)-**7**. The variance values of the absorption dissymmetry factor *g*_abs_ (defined as *g*_abs_ = Δε/ε) are quite small for chiral dinuclear complexes (–)-**3** (−5.1 × 10^−4^ at 316 nm), (–)-**4** (−6.9 × 10^−4^ at 310 nm), (–)-**5** (−7.8 × 10^−4^ at 310 nm), and (–)-**6** (−7.6 × 10^−4^ at 311 nm). The *g*_abs_ for mononuclear Pt(II) complex (–)-**7** also does not differ much, with a value of −5.5 × 10^−4^ at 311 nm. Thus, these bridging ligands (bridging carbon atoms number > 2) have little impact on the CD signals, and it can be inferred that the chiroptical properties of ground electronic states mainly come from an independent [(–)-(C^∧^N^∧^N)Pt]^+^ unit.

**Figure 3:**
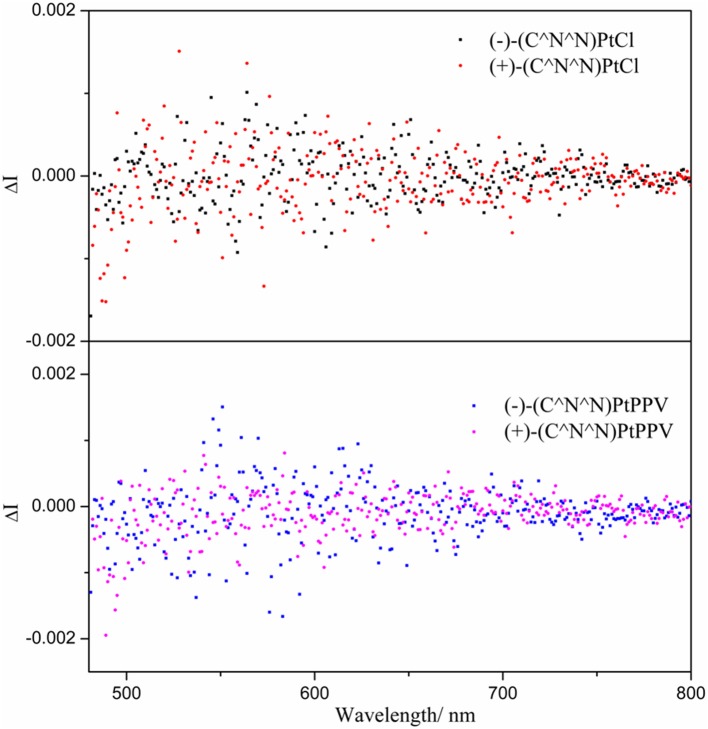
CPL spectra of (–)-(C^∧^N^∧^N)PtCl and (+)-(C^∧^N^∧^N)PtCl, (–)-(C^∧^N^∧^N)PtPPV and (+)-(C^∧^N^∧^N)PtPPV in CH_2_Cl_2_ (10^−3^ mol L^−1^) at *T* = 298 K.

In the precursor monomer Pt(II) complexes, the chiral block pinene substituent prohibits either a helical or axial geometry; its contribution to chirality-at-metal is also limited. Correspondingly, the CPL activity of the monomer state of chiral Pt(II) complexes grafted with pinene groups was very weak, with low *g*_lum_ values (~10^−4^ order), and the mirror-imaged CPL spectra could not be obtained (Zhang et al., 2015a; Lu et al., 2017). The bulky bis- or triphenylphosphine ligands selected in this study both adopt a distorted square-planar coordination of Pt(II), as evidenced in their X-ray-determined crystal structures. As expected, the CPL signals of chiral dinuclear Pt(II) complexes exhibit almost mirror image spectra with respect to their enantiomers (complex **3** in Figure 2 and complexes **4**–**6** in Figures S2–S4). The *g*_lum_ values around the maximum emission wavelength are −1.5 × 10^−3^/+1.2 × 10^−3^ for (–)-**3**/(+)-**3** (Figure 2), −1.2 × 10^−3^/+1.6 × 10^−3^ for (–)-**4**/(+)-**4**, −1.4 × 10^−3^/+1.0 × 10^−3^ for (–)-**5**/(+)-**5**, and −1.3 × 10^−3^/+1.0 × 10^−3^ for (–)-**6**/(+)-**6** (Figures S2–S4). These are comparable to the values reported for helicene- and binaphthyl-derived Pt(II) complexes or helical assemblies of square-planar Pt(II) complexes, with CPL values from 10^−3^ to 10^−2^ order (Shen et al., 2014; Schulte et al., 2017; Ikeda et al., 2018; Song et al., 2019). Similarly, CPL signals can be unambiguously detected for mononuclear complexes (–)-**7** and (+)-**7** with opposite *g*_lum_ values [(–)-**7**: −1.0 × 10^−3^; (+)-**7**: +1.0 × 10^−3^ at 546 nm) (Figure 2). A comparison of the CPL measurements for chiral mononuclear complexes (–)-(C^∧^N^∧^N)PtCl and (–)-(C^∧^N^∧^N)PtPPV has been performed. No appreciable CPL activity was detectable (Figure 3). A similar phenomenon has been observed for chiral (–)-(C^∧^N^∧^N)PtDmpi complexes before (Zhang et al., 2015a).

Complexes (–)-**3**, (–)-**4**, (–)-**5**, (–)-**6**, and (–)-**7** show negative CPL activity; similar spectra have been observed for (–)-**2**. Therefore, the chiroptical properties of excited states mainly originate from monomeric ^3^MLCT (Zhang et al., 2018). Unlike the change of CPL activity induced by the Pt···Pt interaction, π–π stacking effects have little influence on the CPL signals in this system. The CPL activity mostly derives from discrete molecules as a monomeric form. The incorporation of bulky ligands with steric hindrance favors a more distorted coordination geometry for central Pt atoms and enlarges the asymmetry at the metal center, leading to an enhancement in the CPL activity.

### TD-DFT Calculation

Time-dependent density functional theory (TD-DFT) calculations were carried out, shedding light on the differences in the structural parameters and frontier molecular orbitals of optimized configurations. The optimized configurations of all the chiral dinuclear and mononuclear Pt(II) complexes are shown in Figure S6. Also, the calculated results of the reference mononuclear compounds (–)-(C^∧^N^∧^N)PtCl, (–)-(C^∧^N^∧^N)PtPPV, and (–)-(C^∧^N^∧^N)PtDmpi have been provided. The bond angles around the metal nucleus of chiral Pt(II) complexes coordinated with bis- or triphenylphosphine ligands are further away from linearity than those of the reference mononuclear compounds (Table 2), which is consistent with the results of the crystal structures. In optimized configurations with phosphine ligands, the angles of C1–Pt1–N2 and C2–Pt2–N4 are in the range of 157.10–158.08°, and the angles of N1–Pt1–P1 and N3–Pt2–P2 range from 170.97° to 176.82°. It is further confirmed that the Pt(II) nucleus in bulk bis- or triphenylphosphine systems adopts a more distorted coordination geometry.

**Table 2:** Bond angles around the Pt(II) nucleus of the opitimized configurations obtained from calculation.

**Bond angles**	**(–)-1**	**(–)-2**	**(–)-3**	**(–)-4**	**(–)-5**	**(–)-6**	**(–)-7**	**(–)-(C^**∧**^N^**∧**^N)PtCl**	**(–)-(C^**∧**^N^**∧**^N)PtPPV**	**(–)-(C^**∧**^N^**∧**^N)PtDmpi**
C1–Pt1–N2	157.69	158.08	157.76	157.70	157.42.	157.76				
C2–Pt2–N4	157.10	158.03	157.85	157.64	157.76	157.37				
N1–Pt1–P1	175.38	171.59	176.82	173.69	171.80	175.81				
N3–Pt2–P2	176.41	171.67	175.85	174.69	176.14	170.97				
C1–Pt1–N2							157.60	160.52	158.95	159.37
N1–Pt1–P1 (Cl1, C2)							175.42	177.30	178.82	178.52

**Figure 4:**
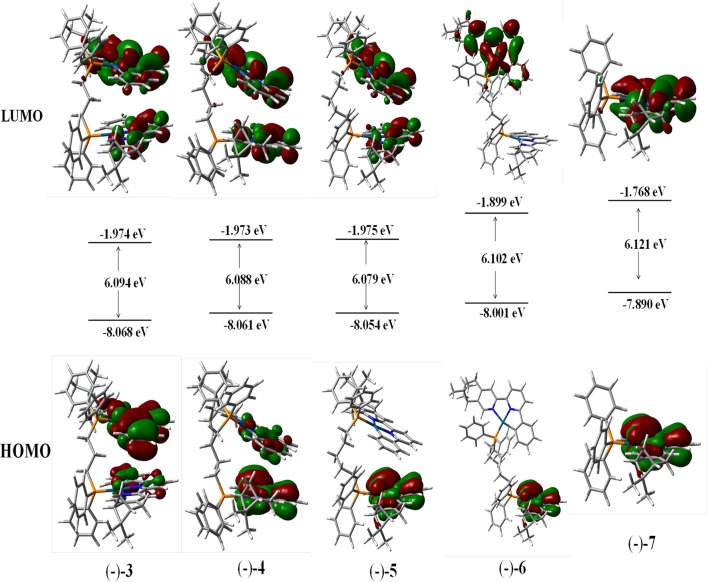
Contour plots of the highest occupied (HOMO) and lowest unoccupied (LUMO) molecular orbitals of (–)-**3**, (–)-**4**, (–)-**5**, (–)-**6**, and (–)-**7**.

The calculations of the frontier molecular orbitals of (–)-**3**, (–)-**4**, (–)-**5**, (–)-**6**, and (–)-**7** have been explored. The highest occupied molecular orbital (HOMO) and the lowest unoccupied molecular orbital (LUMO) of all the complexes are mainly contributed by the aromatic rings of (–)-(C^∧^N^∧^N) ligands and Pt atoms (Figure 4). Due to the longer bridging ligands, neither obvious bonding orbitals between the two Pt centers nor bonding orbitals between the two aromatic C^∧^N^∧^N planes can be visualized in (–)-**3**, (–)-**4**, (–)-**5**, and (–)-**6** (Figure 4), while strong and weak bonding orbitals between the two Pt centers are visible in complexes (–)-**1** and (–)-**2**, respectively (Zhang et al., 2018). The HOMO–LUMO band gaps of (–)-**3**, (–)-**4**, (–)-**5**, (–)-**6**, and (–)-**7** are calculated to be 6.094, 6.088, 6.079, 6.102, and 6.121 eV, respectively (Figure 4), revealing that the bridging ligands (bridging carbon atoms > 2) cause little difference in band gaps. In addition, the CD spectra have been simulated, and the computed spectra in dichloromethane are in good agreement with the experimental profiles (Figures S7–S11).

## Conclusion

In summary, we introduced bulky bis- or triphenylphosphine ligands into the phosphorescent pinene-containing (C^∧^N^∧^N)Pt(II) complexes and their structures were determined by single-crystal X-ray analysis. The geometries around the Pt(II) nucleus upon coordinating with bis- or triphenylphosphine were more distorted than those in chloride, phenylacetylene, and 2,6-dimethylphenyl isocyanide systems, which was further verified by DFT calculations. Enhanced CPL activity was observed, with *g*_lum_ up to 10^−3^ order. This study may pave a new way for the preparation of CPL-active phosphorescent metal complexes by introducing bulky ligands.

## Data Availability Statement

The datasets generated for this study can be found in the Cambridge Crystallographic Data Centre (https://www.ccdc.cam.ac.uk/structures/) under the identifiers 1984372-1984374.

## Author Contributions

The preparation and characterization of all the complexes were done mainly by Q-YY, X-LH, and J-LW. The spectra measurement was done mainly by Q-YY, H-HZ, and YC. The TD-DFT calculation was done mainly by S-DW, L-ZH, and Z-FS. The manuscript was written by Q-YY with the guidance of X-PZ.

## Conflict of Interest

The authors declare that the research was conducted in the absence of any commercial or financial relationships that could be construed as a potential conflict of interest. The handling editor declared a past co-authorship with one of the authors X-PZ.
