# Supplementary material for: Enhanced Circularly Polarized Luminescence Activity in Chiral Platinum(II) Complexes With Bis- or Triphenylphosphine Ligands
Source: Front Chem. 2020 Apr 24;8:303. doi: 10.3389/fchem.2020.00303 (PMC7193082; doi:10.3389/fchem.2020.00303)
Supplement: Supplementary file 1 [file Data_Sheet_1.docx]

**Supporting Information**

**for**

**Enhanced Circularly Polarized Luminescence Activity in Chiral Platinum(II) Complexes with Bis- or Tri-phenylphosphine Ligands**

Qian-Ying Yang ^1^, Hua-Hong Zhang ^1^, Xue-Ling Han ^1^, Shi-Dao Weng ^1^, Yuan Chen ^1^, Jia-Li Wu ^1^, Li-Zhi Han ^1^, Xiao-Peng Zhang ^1*^, Zai-Feng Shi ^1^

^1^ Key Laboratory of Water Pollution Treatment & Resource Reuse of Hainan Province, College of Chemistry and Chemical Engineering, Hainan Normal University, Haikou 571158, China

*** Correspondence:**Xiao-Peng Zhang
zxp_inorganic@126.com

**Materials and methods, crystal structures, spectroscopic properties and TD-DFT calculation results**

**Table of Contents**

1. Materials and Methods .......................................................................................... 2

2. Crystal structures ................................................................................................... 4

3. Spectroscopic properties ........................................................................................ 6

4. TD-DFT calculation results ..................................................................................10

5. References ............................................................................................................ 13

# 1. Materials and Methods

**1.1 General Methods**

All reagents were purchased from commercial suppliers and used as received. Mass spectra were acquired on an LCQ Fleet ESI Mass Spectrometer. The NMR spectra were obtained on Bruker DRX-400 spectrometer. Coupling constants are given in hertz. UV-vis spectra were measured on a UV-3600 spectrophotometer. Elemental analysis was performed on a Perkin-Elmer 240C analyzer. Photoluminescence (PL) spectra were measured by Hitachi F-4600 PL spectrophotometer (λ_ex_ = 420 nm). The circular dichroism (CD) spectra in CH_2_Cl_2_ solution were recorded on a Jasco J-810 spectropolarimeter at a scan rate of 100 nm·min^−1^ and 1 nm resolution at room temperature (using 10 mm quartz cell for concentration of 5×10^-5^ mol·L^-1^, bandwidth = 1 nm, response = 1 sec, accumulations = 3). The CPL spectra in CH_2_Cl_2_ solution were recorded a Jasco CP-300 spectrofluoropolarimeter at a scan rate of 100 nm·min^−1^ and 1 nm resolution at room temperature (using 10 mm quartz cell for concentration of 10^-3^ mol·L^-1^, E_x_ wavelength = 330 nm, D.I.T. = 2 sec, E_x_ slit width = 4000 μm, Em slit width = 4000 μm, Detector: PM-539, Detector S/N: B0005PM539, Lock-in amp.: X mode, HT volt = 670-720V, Accessory: RSC-502, Accessory S/N: A0005RSC502, accumulations = 10).

**1.2 Single Crystal X-ray Structure Determination**

Single-crystal X-ray diffraction measurements were carried out on a Bruker SMART APEX CCD based on diffractometer operating at 153 K. Intensities were collected with graphite monochromatized Mo Kα radiation (*λ* = 0.71073 Å) operating at 50 kV and 30 mA, using ω/2θ scan mode. The data reduction was made with the Bruker SAINT package.^[1]^ Absorption corrections were performed using the SADABS program.^[2]^ The structures were solved by direct methods and refined on F^2^ by full-matrix least-squares using SHELXL-97 with anisotropic displacement parameters for all non-hydrogen atoms in all two structures. Hydrogen atoms bonded to the carbon atoms were placed in calculated positions and refined as riding mode, with C−H = 0.93 Å (methane) or 0.96 Å (methyl) and Uiso(H) = 1.2Ueq (C_methane_) or Uiso(H) = 1.5Ueq (C_methyl_). The water hydrogen atoms were located in the difference Fourier maps and refined with an O-H distance restraint [0.85(1) Å] and Uiso(H) = 1.5 Ueq(O). All computations were carried out using the SHELXTL-97 program package.^[3]^ CCDC 1984372-1984374 contain the supplementary crystallographic data for this paper. These data can be obtained free of charge from The Cambridge Crystallographic Data Centre via www.ccdc.cam.ac.uk/data_request/cif.

**1.3 Calculation Methods**

Crystal structures (−)-**3**-OTf, (−)-**4** and (−)-**7** were used as the starting geometry, and optimization was performed by the Gaussian09 program.^[4]^ The starting structures of (−)-**5** and (−)-**6** are based on the crystal structure (−)-**3**-OTf that adding one carbon atom on the bridging ligands. The CAM-B3LYP functional was used, SVP basis set for C, H, N, and P, and MWB60 pseudopotential basis set for Pt, and the conductor-like polarizable continuum solvent model (CPCM).^[5]^ TD-DFT simulations was performed with same functional and basis sets. The calculated CD spectrum was drawn by using the software SpecDis,^[6]^ in which the sigma/eV was set at 0.2.

# 2. Crystal structures

**Table S1** Bond lengths of complexes (−)-**3**-OTf, (−)-**4** and (−)-**7** determined by X-ray single crystal diffraction.

| Bond Lengths | (−)-**3**-OTf | (−)-**4** | (−)-**7** |
| --- | --- | --- | --- |
| Pt1−C1 | 2.016(5) | 1.972(7) | 2.043(7) |
| Pt2−C2 | 2.007(6) | 1.994(9) | 2.025(7) |
| Pt1−N1 | 1.985(5) | 1.998(5) | 2.0175(18) |
| Pt1−N2 | 2.119(5) | 2.167(6) | 2.135(6) |
| Pt2−N3 | 1.984(6) | 1.973(6) | 2.014(6) |
| Pt2−N4 | 2.108(4) | 2.104(7) | 2.143(6) |
| Pt1−P1 | 2.2478(17) | 2.2503(18) | 2.2445(19) |
| Pt2−P2 | 2.245(2) | 2.241(2) | 2.2522(19) |
| Pt3−C3 |  | 2.019(8) |  |
| Pt4−C4 |  | 2.006(8) |  |
| Pt3−P3 |  | 2.259(2) |  |
| Pt4−P4 |  | 2.2351(19) |  |
| Pt3−N5 |  | 1.998(6) |  |
| Pt3−N6 |  | 2.146(6) |  |
| Pt4−N7 |  | 2.016(6) |  |
| Pt4−N8 |  | 2.118(6) |  |

**Table S2** Bond angles around Pt(II) nucleus of complexes (−)-**3**-OTf, (−)-**4**, (−)-**7** and reported (−)-(C^N^N)PtCl, (−)-(C^N^N)PtPPV, (−)-(C^N^N)PtDmpi.

| Bond Angles | (−)-**3**-OTf | (−)-**4** | (−)-**7** | (C^N^N)PtCl | (C^N^N)Pt PPV- Form-Y | (C^N^N)PtPPV- Form-O | (C^N^N)Pt Dmpi- Form-Y | (C^N^N)PtDmpi- Form-R |
| --- | --- | --- | --- | --- | --- | --- | --- | --- |
| C1−Pt1−N2 | 158.8(2) | 159.4(3) | 158.9(3) . | 162.2(4) | 160.75(16) | 160.88(15) | 160.1(10) | 162.3(4) |
| N1−Pt1−P1 (Cl1, C2) | 176.10(16) | 171.79(18) | 174.51(10) | 178.0(2) | 176.7(2) | 178.23(15) | 177.6(12) | 175.3(4) |
| C2−Pt2−N4 | 158.7(3) | 157.9(3) | 159.0(3) | 162.4(5) |  |  | 161.2(5) |  |
| N3−Pt2−P2 (Cl2, C4) | 173.60(15) | 168.75(17) | 174.6(2) | 178.2(3) |  |  | 178.2(13) |  |
| C3−Pt3−N6 |  | 157.3(3) |  |  |  |  |  |  |
| N5−Pt3−P3 |  | 173.57(18) |  |  |  |  |  |  |
| C4−Pt4−N8 |  | 158.3(3) |  |  |  |  |  |  |
| N7−Pt4−P4 |  | 169.63(19) |  |  |  |  |  |  |

**Table S3** Torsion angles between the benzene plane and lateral pyridine plane of C^N^N ligands in complexes (−)-**3**-OTf, (−)-**4** and (−)-**7**.

| Torsion angles | (−)-**3**-OTf | (−)-**4** | (−)-**7** |
| --- | --- | --- | --- |
| benzene(1) − pyridine(1) | 7.281(153) | 5.191(216) | 8.160(289) |
| benzene(2) − pyridine(2) | 8.224(183) | 11.209(250) | 1.333(312) |
| benzene(3) − pyridine(3) |  | 14.514(265) |  |
| benzene(4) − pyridine(4) |  | 9.412(232) |  |

**Figure S1** Intermolecluar Pt⋅⋅⋅Pt contacts of complex (−)-**3**-OTf, (−)-**4** and (−)-**7**. H atoms and anions are omitted for clarity.

**2. Spectroscopic properties**

**Figure S2** Absorption spectra and emission spectrum of (−)-**4** in CH_2_Cl_2_ (5×10^-5^ mol·L^-1^) at T = 298 K, *λ*_ex_ = 420 nm (top); CD spectra in CH_2_Cl_2_ (5×10^-5^ mol·L^-1^) at T = 298 K (middle); CPL spectra in CH_2_Cl_2_ (10^-3^ mol·L^-1^) at T = 298 K (middle); *g*_abs_ and *g*_lum_ factors (bottom).

**Figure S3** Absorption spectra and emission spectrum of (−)-**4** in CH_2_Cl_2_ (5×10^-5^ mol·L^-1^) at T = 298 K, *λ*_ex_ = 420 nm (top); CD spectra in CH_2_Cl_2_ (5×10^-5^ mol·L^-1^) at T = 298 K (middle); CPL spectra in CH_2_Cl_2_ (10^-3^ mol·L^-1^) at T = 298 K (middle); *g*_abs_ and *g*_lum_ factors (bottom).

**Figure S4** Absorption spectra and emission spectrum of (−)-**4** in CH_2_Cl_2_ (5×10^-5^ mol·L^-1^) at T = 298 K, *λ*_ex_ = 420 nm (top); CD spectra in CH_2_Cl_2_ (5×10^-5^ mol·L^-1^) at T = 298 K (middle); CPL spectra in CH_2_Cl_2_ (10^-3^ mol·L^-1^) at T = 298 K (middle); *g*_abs_ and *g*_lum_ factors (bottom).

**Figure S5** Emission spectra of (−)-**3**, (−)-**4**, (−)-**5**, (−)-**6** and (−)-**7** at 298 (solid line) and 77 K (dash line) (*λ*ex = 420 nm).

**2. TD-DFT calculation results**

**Figure S6** The optimized configurations of all the chiral dinuclear and mononuclear Pt(II) complexes.

**Figure S7** Simulated rotatory strength (green), and CD spectrum (red) of complex (−)-**3** in dichloromethane solution in comparison with its experimental one (black).

**Figure S8** Simulated rotatory strength (green), and CD spectrum (red) of complex (−)-**4** in dichloromethane solution in comparison with its experimental one (black).

**Figure S9** Simulated rotatory strength (green), and CD spectrum (red) of complex (−)-**5** in dichloromethane solution in comparison with its experimental one (black).

**Figure S10** Simulated rotatory strength (green), and CD spectrum (red) of complex (−)-**6** in dichloromethane solution in comparison with its experimental one (black).

**Figure S11** Simulated rotatory strength (green), and CD spectrum (red) of complex (−)-**7** in dichloromethane solution in comparison with its experimental one (black).

**5. References**

[1] *SAINT-Plus*, version 6.02, Bruker Analytical X-ray System: Madison, WI, 1999.

[2] G. M. Sheldrick, *SADABS*, an empirical absorption correction program, Bruker Analytical X-ray Systems: Madison, WI, 1996.

[3] G. M. Sheldrick, *Acta Cryst.*, 2008, A**64**, 112.

[4] Frisch, M. J. T., G. W.; Schlegel, H. B.; Scuseria, G. E.; Robb, M. A.; Cheeseman, J. R.; Scalmani, G.; Barone, V.; Mennucci, B.; Petersson, G. A.; Nakatsuji, H.; Caricato, M.; Li, X.; Hratchian, H. P.; Izmaylov, A. F.; Bloino, J.; Zheng, G.; Sonnenberg, J. L.; Hada, M.; Ehara, M.; Toyota, K.; Fukuda, R.; Hasegawa, J.; Ishida, M.; Nakajima, T.; Honda, Y.; Kitao, O.; Nakai, H.; Vreven, T.; Montgomery, J. A., Jr.; Peralta, J. E.; Ogliaro, F.; Bearpark, M.; Heyd, J. J.; Brothers, E.; Kudin, K. N.; Staroverov, V. N.; Kobayashi, R.; Normand, J.; Raghavachari, K.; Rendell, A.; Burant, J. C.; Iyengar, S. S.; Tomasi, J.; Cossi, M.; Rega, N.; Millam, J. M.; Klene, M.; Knox, J. E.; Cross, J. B.; Bakken, V.; Adamo, C.; Jaramillo, J.; Gomperts, R.; Stratmann, R. E.; Yazyev, O.; Austin, A. J.; Cammi, R.; Pomelli, C.; Ochterski, J. W.; Martin, R. L.; Morokuma, K.; Zakrzewski, V. G.; Voth, G. A.; Salvador, P.; Dannenberg, J. J.; Dapprich, S.; Daniels, A. D.; Farkas, O.; Foresman, J. B.; Ortiz, J. V.; Cioslowski, J.; Fox, D. J. Gaussian 09, Gaussian, Inc.: Wallingford CT, 2013.

[5] Klamt, A., *J. Phys. Chem.*, 1995, **99**, 2224-2235.

[6] Bruhn, T., Schaumlöffel, A., Hemberger, Y., *SpecDis version 1.63*, University of Wuerzburg, Germany, 2015.
